# Multilevel factors associated with sleep duration and bedtime regularity in U.S. children with and without neurodevelopmental disorders: a nationally representative study

**DOI:** 10.1007/s12519-025-00964-w

**Published:** 2025-10-03

**Authors:** Freda Patterson, Shannon M Robson, Lauren B Covington, Carissa M Baker-Smith, Shannon Mayberry, Ben Brewer, Zugui Zhang, Anjana Bhat

**Affiliations:** 1 Department of Health Behavior and Nutrition Sciences, College of Health Sciences, University of Delaware, 100 Discovery Blvd, Newark, DE 19716, USA; 2 Cardiovascular Research and Innovation Program, Nemours Cardiac Center, Nemours Children’s Health, Wilmington, DE, USA; 3 Institute for Research On Equity and Community Health, Christiana Care Health System Inc, Newark, DE, USA; 4 Department of Physical Therapy, College of Health Sciences, University of Delaware, Newark, DE, USA; 5 School of Nursing, College of Health Sciences, University of Delaware, Newark, USA; 6 Biostatistics Core, College of Health Sciences, University of Delaware, Newark, USA

**Keywords:** Bedtime regularity, Children, Machine learning, Neurological and developmental disorders, Sleep duration

## Abstract

**Background:**

This study compared multilevel factors associated with sleep duration and bedtime regularity in children with and without neurological and developmental disorders (NDD) using a nationally representative sample.

**Methods:**

We combined data from the 2016–2017, 2018–2019, and 2020–2021 United States National Survey of Children's Health (NSCH), including 86,014 children aged 9–17 years. Parent-reported outcomes included adequate sleep duration (yes/no) and bedtime regularity (yes/no). We assessed the relationship between twenty independent individual, social, and environmental variables on the sleep outcomes. We used random survival forest decision trees to identify the five most predictive variables [in terms of variable importance (VIMP)]. Regression analyses were then used to assess directionality and independent associations.

**Results:**

A greater proportion of children with NDDs versus those who are typically developing (TD) had parent reports of not achieving adequate sleep duration (34.0% versus 30.9%, respectively) and bedtime regularity (18.8% versus 13.7%, respectively). For adequate sleep, achieving bedtime regularity, having more family meals together and older age emerged as the most important independent variables for both TD children and those with NDDs. For bedtime regularity, achieving adequate sleep, and having more family meals together were the most proximal independent variables for NDD and TD children.

**Conclusion:**

Increasing the frequency of family meals and addressing both sleep duration and bedtime regularity may serve as key modifiable intervention targets to improve sleep health in children, including those with NDDs.

## Graphical Abstract

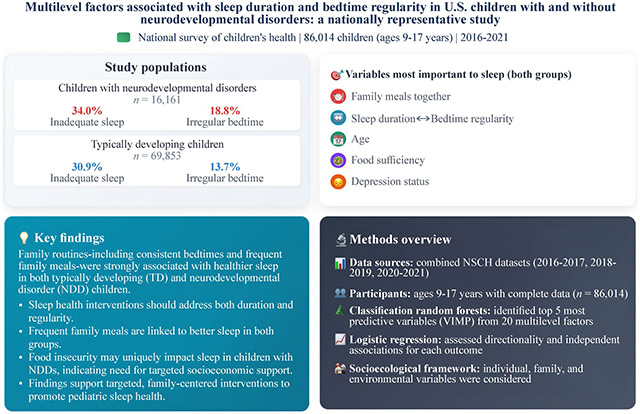

## Introduction

Healthy sleep is a biological necessity for pediatric populations, essential to the optimal development and functioning of all bodily systems including cognitive and neurological, immunological, cardiovascular and metabolic systems [1]. Children with neurodevelopmental disorders (NDDs)—conditions that manifest in early childhood due to central nervous system injury and impair one or more areas of functioning (e.g., physical or motor, social, emotional, behavioral) [2]—are substantially more likely to experience sleep disturbances [3, 4]. Up to 75% of children with an NDD report short and/or irregular sleep as compared to 30% of typically developing (TD) children in the general population [5-7].

There are several reasons to explain why children with NDDs experience poorer sleep. First, many NDDs involve disruptions to neural circuits that regulate circadian rhythms and sleep–wake cycles, including alterations in melatonin production, neurotransmitter function and brain regions responsible for sleep regulation [8]. For example, children with autism spectrum disorder often show atypical melatonin secretion patterns, while those with ADHD frequently exhibit delayed sleep phase syndrome [9, 10]. Second, core symptoms of NDDs can directly interfere with sleep initiation and maintenance. Hyperactivity and impulsivity in ADHD, repetitive behaviors and sensory sensitivities in autism, and motor symptoms in cerebral palsy can all disrupt bedtime routines and sleep quality [11]. Additionally, medications commonly prescribed for NDDs, including stimulants and antipsychotics, can significantly impact sleep architecture and timing [12]. Third, children with NDDs often experience higher rates of comorbid conditions that further compromise sleep, including anxiety, depression, gastrointestinal issues, and seizure disorders [13]. These comorbidities create a complex web of factors that can perpetuate sleep disturbances.

While the association between sleep and development is complex, achieving the recommended hours of sleep on a regular basis is reliably associated with better emotional, neurobehavioral, neurocognitive and cardiometabolic outcomes in both typically developing (TD) children and those with NDDs [14]. However, the consequences of poor sleep may be particularly severe for children with NDDs. Sleep deprivation can impede cognitive function and learning abilities [15]. In children with NDDs, poor sleep health can exacerbate core symptoms of their condition, creating a cycle where NDD symptoms worsen sleep, which in turn intensifies NDD-related difficulties [16, 17]. Another critical consideration in the context of poor sleep health in children is the negative repercussions for family well-being and functioning. In families with children reporting disordered sleep, the likelihood of parental stress is significantly increased and family functioning decreased [18, 19]. This family-level impact underscores the importance of addressing sleep as a modifiable target that can improve outcomes for both the child and the broader family system.

Despite the well-documented sleep disparities between NDD and TD populations, significant gaps remain in our understanding of the multilevel factors that predict sleep outcomes in these groups. The socioecological framework of sleep health posits that individual, family, neighborhood, and community-level factors interact to determine sleep duration and timing [20]. At the individual level, demographic characteristics (age, sex, race/ethnicity) and health behaviors (physical activity, screen time) have been consistently shown to predict sleep health metrics in pediatric populations [21]. Family-level variables include family structure (household composition, parental marital status), family functioning indicators (family meals, family resilience), and socioeconomic factors (income, parental education, employment) that research has linked to sleep health in children [22]. Similarly, community and environmental factors including neighborhood characteristics (safety, social cohesion, amenities) and healthcare access have been shown to influence sleep health in children [23]. To date, this previous research has focused on clinical samples or single diagnostic categories, limiting generalizability to the broader NDD population [24]. Additionally, few studies have systematically examined whether the same predictive factors influence sleep outcomes similarly across NDD and TD groups [25], or whether different intervention targets may be needed for these populations.

Given these knowledge gaps, the primary goal of this study was to use machine learning methods to identify the strongest multilevel predictors of adequate sleep duration and bedtime regularity in a nationally representative sample of TD children and those with NDDs. Multiple variables representing individual, family, and community levels of the socioecological framework were examined to determine whether predictive patterns differ between groups. Results of this theoretically and empirically guided investigation are expected to identify modifiable intervention targets that can improve sleep duration and regularity for both NDD and TD children.

## Methods

Data for this study were collected from the National Survey of Children’s Health (NSCH), an annual household survey by the U.S. Census Bureau assessing the health and well-being of non-institutionalized children ages 0–17 years. U.S. households with children are randomly selected from a nationwide address list. A screener determines eligibility, and one child is randomly selected for an age-specific survey (0–5, 6–11, or 12–17 years). A parent or guardian familiar with the child’s condition completes the survey, which can be administered via mail, phone, or online. Publicly available data from the 2016–2017, 2018–2019, and 2020–2021 datasets were used. Weighted response rates were: 40.7% (2016), 37.4% (2017), 43.1% (2018), 42.4% (2019), 42.4% (2020), and 40.3% (2021). Analyses were weighted for selection and nonresponse bias. Detailed survey design and ethical approval information are available elsewhere [26].

## Study participants

Participants in this study were aged 9–17 years and had complete data for study variables. The omnibus dataset with the combined datasets from years 2016–2017, 2018–2019, and 2020–2021 had 123,215 cases. Including children beginning at age 9 years in the analytic sample maximized the likelihood that the presence and subsequent diagnosis of any neurological and developmental diagnosis would have been made since diagnosis by age 8 years is likely [27]. Participants with missing data for any of the study variables (*n* = 37,201) were excluded, yielding a final analytic sample of 86,014. Participants with missing data on any of the study variables did not significantly differ demographically (sex and race) from the analytic sample, suggesting minimal selection bias on these key characteristics.

### Study measures

#### Dependent variables

Adequate sleep duration: Parent or guardians reported the average hours of sleep that their child received on most weeknights in the past week (< 6, 6, 7, 8, 9, 10 hours, or ≥ 11 hours). Based on age-appropriate sleep duration recommendations [28] that suggest 9–12 hours/day of sleep for ages 9–12 years and 8–10 hours for ages 13–17 years, responses were coded to group children into adequate sleep duration “Yes” (sleeps recommended hours) versus “No” (does not sleep recommended hours) categories.

Bedtime regularity: Parent or guardians answered the single item, “how often does this child go to bed at about the same time on weeknights?” Response options were on a 4-point scale: always, usually, sometimes, and rarely/never and dichotomized such that “always” and “usually” responses were considered as “Yes” (has regular bedtimes) and “sometimes” or “rarely/never” as “No” (does not have regular bedtimes).

#### Independent variables

Independent variables were grouped in accordance with the levels of the social ecological model: individual, social, and environmental.

#### Individual

During the screening questionnaire, parents or guardians reported their child’s age, sex (male/female), and race/ethnicity (Hispanic, White non-Hispanic, Black non-Hispanic, or other/multiracial non-Hispanic).

#### Family income level

Parents or guardians reported household income in the last calendar year. Responses were grouped as follows based on the percentage of the federal poverty level (FPL): (1) 0%–99% FPL, (2) 100%–199% FPL, (3) 200%–399% FPL, and (4) 400% FPL or greater. Higher scores indicate greater poverty.

##### Neurodevelopmental disorder (NDD) status

Guided by DSM-V criteria, children reported to currently have one or more of the following NDDs were classified as having a NDD: Autism, ADHD, Down syndrome, intellectual disability, learning disability, Tourette syndrome, or speech/other language disorder. Children not reported as having any of these conditions were categorized as typically developing (TD).

##### Physical activity (PA)

Parents/guardians reported how many days in the past week the child exercised, played sports, or was active for at least 60 min. Response options: (1) 0 days, (2) 1–3 days, (3) 4–6 days, (4) every day.

##### Screen time

Parents/guardians reported weekday hours spent watching TV/videos, playing video games, or using electronics (excluding schoolwork). Responses: (1) < 1 hour/day, (2) 1–2 hours/day, (3) ≥ 3 hours/day.

##### Ever and current depression:

Parents indicated whether a healthcare provider had ever diagnosed their child with depression (1 = yes; 0 = no). If yes, they were asked if the child currently had depression (1 = yes; 0 = no).

#### Social level variables

##### Maternal mental health

Mothers rated their mental/emotional health on a 3-point scale (1 = excellent/very good, 2 = good, 3 = fair/poor), with higher scores indicating poorer health.

##### Family meal frequency

Parents/guardians reported how many days in the past week household members ate a meal together. Responses: (1) 0 days, (2) 1–3 days, (3) 4–6 days, (4) every day.

##### Difficulty making/keeping friends

Parents rated the child’s difficulty making or keeping friends on a 3-point scale (1 = no difficulty, 2 = a little difficulty, 3 = a lot of difficulty).

##### Being bullied

Parents or guardians reported if their child was bullied, picked on, or excluded by other children in the last year (yes/no).

##### Parental aggravation

On a 5-point scale (Never–Always), parents answered three questions: (1) Is this child harder to care for than others their age? (2) Does this child often bother you? (3) Are you often angry with this child? If “usually” or “always” was selected for any item, parents were categorized as “usually/always aggravated” (1); all others as (2) “seldom aggravated.”

#### Environmental level variables

Someone smokes inside the home: Parents reported whether someone used tobacco products inside the home (yes/no).

Food sufficiency: Parents answered how well they could afford food over the past 12 months using a 4-point scale: (1) always enough nutritious food, (2) always enough but not the right food, (3) sometimes not enough, (4) often not enough.

Neighborhood safety: Parents rated the statement “Child is safe in this neighborhood” on a 3-point scale (1 = definitely agree, 2 = somewhat agree, 3 = somewhat/definitely disagree), with lower scores indicating greater safety.

Detracting neighborhood elements: Parents or guardians reported whether the following three items were present in their neighborhood: litter or garbage on the streets/sidewalk, poorly kept housing, or vandalism (broken windows/graffiti). Answers ranged from zero to three detracting elements.

Neighborhood social support: Parents indicated agreement with three statements: (1) neighbors help each other, (2) watch out for each other’s children, (3) know where to go for help. Respondents were considered to live in supportive neighborhoods if parents “definitely agreed” with at least one and “somewhat/definitely agreed” with the others.

Neighborhood amenities: This measure is a count of how many of the following four (parent/guardian reported) amenities are present in the child’s neighborhood: sidewalks/walking paths, park/playground, recreation center/boys’ and girls’ club, or libraries/bookmobiles.

Access to family centered care: Parents of children who had a health visit in the past year reported on five experience-of-care items: provider spends time, listens, respects values, provides information, and treats family as partner. “Usually” or “always” on at least one item indicated receipt of family-centered care (1 = yes).

### Statistical analysis

Descriptive statistics were generated for all study variables. Unadjusted associations between independent variables and each of the study outcomes (i.e., adequate sleep and bedtime regularity) were calculated. To determine the most important variables related to each of the outcomes (adequate sleep duration, bedtime regularity), classification random forests (CRF) were used.

CRFs are a non-parametric ensemble supervised learning method that operate by constructing a multitude of decision trees and then using the individual tree predictions to produce a final outcome prediction through majority vote. CRFs offer several key advantages over traditional regression approaches: they can handle large numbers of variables without overfitting concerns, automatically detect complex associations between variables without requiring a priori specification and are robust to outliers and missing data. To ensure that the individual predictions do not become correlated, each tree utilizes random sampling without replacement to select a subset of variables before splitting a node. Random sampling (with replacement in this case) is also used to select a training set for each tree. Upon completion of the modeling process, it is possible to “rank” the variables used to grow a given CRF by using each variable’s predictive utility, often referred to as variable importance (VIMP). VIMP is a relative measure with higher values indicating a higher level of importance in producing an accurate prediction of the outcome. When a variable with a low VIMP is removed from its model, the veracity of the model’s final prediction will not be significantly impacted. Conversely, when variables with high VIMPs are removed, the model’s final predictions are much more noticeably (and negatively) impacted.

We selected CRFs over alternative ensemble methods (e.g., XGBoost) because our primary objective was variable selection rather than predictive accuracy optimization. CRFs provide interpretable variable importance measures, stable rankings across different hyperparameter settings, and robust performance with mixed data types, making them optimal for identifying intervention targets in explanatory research rather than developing clinical prediction models.

A total of four CRFs were generated—one for each of the sleep health outcomes for NDD and TD children. To compare values more easily across variables, raw VIMP values were first multiplied by 100 before presentation. Further, all VIMPs were reported using plots of 95% confidence intervals (CI), which were in turn approximated through simulations employing bootstrapping and approximately 160,000 individual decision trees per CRF. Since VIMPs do not provide a sense of directionality, we generated separate logistic regression models for each of the CRFs. Independent variables included in each of the logistic regression models were those identified by the regression random forest model as having a high VIMP, defined here as having one of the top 5 highest VIMPs for the respective outcome/cohort pairing. The odds ratios with accompanying 95% CI’s were reported in the logistic regression model results.

All analyses were performed using R v.4.3.1. For the logistic regression results, confidence intervals not containing 1 were statistically significant. The *randomForestSRC* package was used to fit the random forests and extract VIMPs. The function parameters used to grow the forests were set equal to the vignette default values as conceived by the package developers.

## Results

### Sample characteristics

All sample characteristics are shown in Tables 1 and 2 (and supplemental Tables 1 and 2). Among children with a parent-reported NDD (*n* = 16,161), 34.0% reported not having adequate sleep, and 18.8% did not report a regular bedtime. Among TD children (*n* = 69,853), 30.9% did not have adequate sleep, and 13.7% did not report a regular bedtime.

## Bivariate associations between study variables with adequate sleep and bedtime regularity

Given the large sample size and statistical power to detect significance, we emphasize only associations with moderate effect sizes (Cohen’s d ≥ 0.3). In both NDD and TD groups, bedtime regularity was moderately and significantly associated with adequate sleep duration (Tables 1 and 2).

For children with NDDs, the following were moderately and significantly associated with bedtime regularity: adequate sleep, younger age, food sufficiency, physical activity, lower screen time, absence of depression, better maternal mental health, and frequent family meals (Table 1). Among TD children, similar factors were associated with bedtime regularity: adequate sleep, younger age, food sufficiency, physical activity, lower screen time, better maternal mental health, frequent family meals, and living in a supportive neighborhood (Table 2).

## Multivariable associations between study variables with adequate sleep and bedtime regularity

### Adequate sleep duration in children with neurodevelopmental disorders (NDD)

**Fig. 1:**
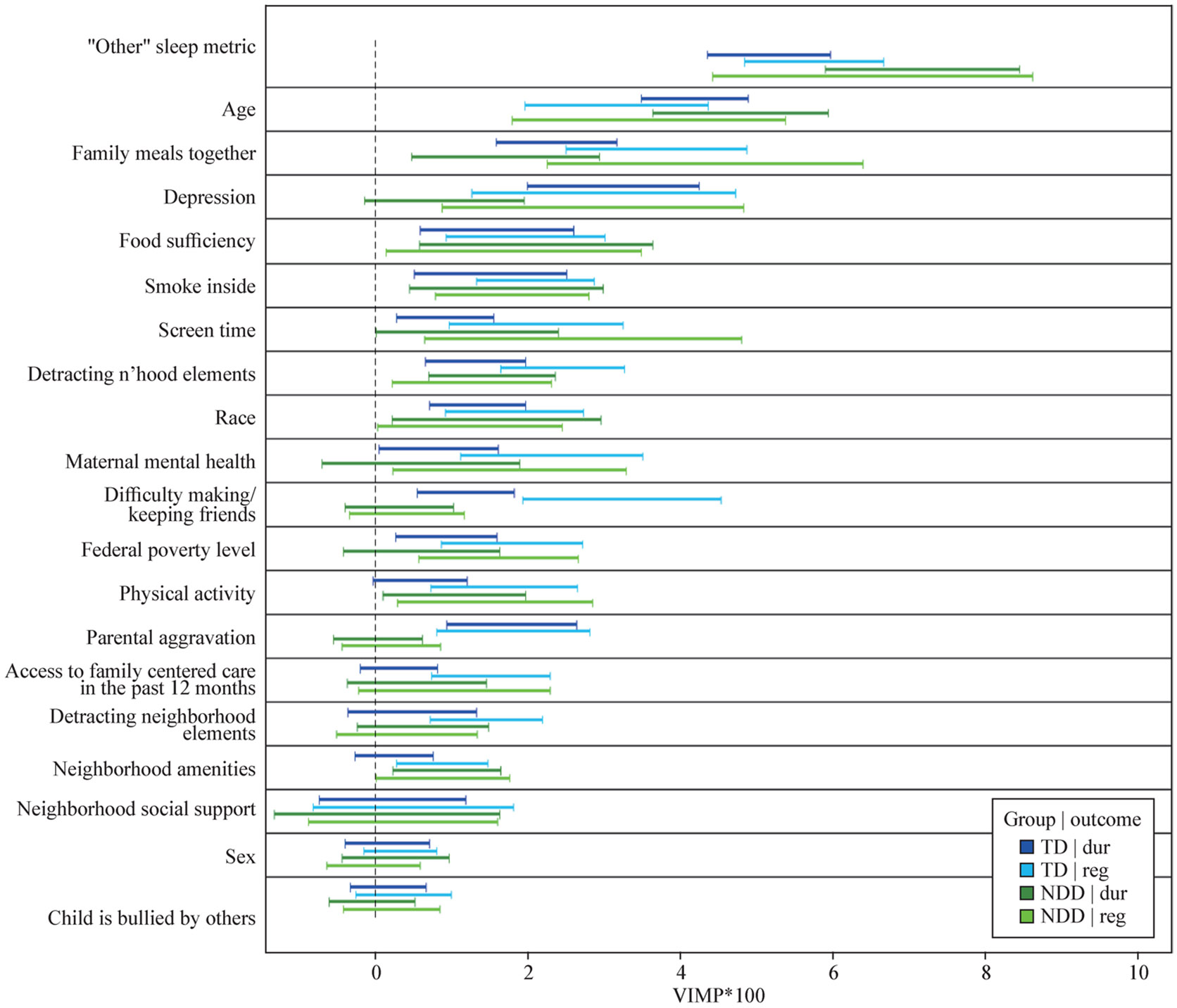
Composite figure illustrating variables of most importance to sleep outcomes for typically developing (TD) children and those with a neurodevelopmental disorder (NDD)

In classification random forests (CRF), key predictors were age (VIMP = 7.79), bedtime regularity (7.17), food sufficiency (2.10), smoking inside home (1.72), and family meals (1.71) (Fig. 1, Supplemental Table S1).

Logistic regression showed that children with regular bedtimes had 2.78 times higher odds of adequate sleep (95% CI = 2.55, 3.02). Those eating family meals daily had 1.63 times the odds (95% CI = 1.40, 1.90). In contrast, children who often could not afford to eat (lowest food sufficiency) had lower odds (OR = 0.69, 95% CI = 0.50, 0.94) of adequate sleep duration (Table 3).

### Adequate sleep duration in typically developing (TD) children

In the CRF model, bedtime regularity (VIMP = 5.16), age (4.19), depression (3.12), family meals (2.37), and parental aggravation (1.79) were key factors (Fig. 1, Supplemental Table 2).

In logistic regression, children with regular bedtimes had 2.40 times greater odds of adequate sleep (95% CI = 2.30, 2.52). Children with daily family meals had 97% higher odds (OR = 1.97, 95% CI = 1.80, 2.15) of adequate sleep (Table 3).

In comparing the factors independently associated with adequate sleep across both groups, the regression models revealed similar patterns: bedtime regularity and daily family meals were significantly associated with higher odds of adequate sleep in both NDD and TD children. However, the magnitude of these associations differed. Among children with NDDs, the association was stronger for regular bedtimes (OR = 2.78 vs. 2.40), whereas TD children showed a stronger association for daily family meals (OR = 1.97 vs. 1.63). Notably, food insufficiency was significantly associated with lower odds of adequate sleep only among children with NDDs (OR = 0.69), highlighting a potential area of increased vulnerability in this group.

### Bedtime regularity in children with neurodevelopmental disorders

In the CRF model, factors most important to bedtime regularity for children with NDDs were adequate sleep duration (VIMP = 6.52), age (VIMP = 3.59), depression (2.85), screen time (VIMP = 2.72) and family meals (VIMP = 2.37) (Fig. 1, Supplemental Table 3).

Logistic regression showed children with adequate sleep had 2.86 times higher odds of regular bedtime (95% CI = 2.63, 3.12). Daily family meals increased odds nearly threefold (OR = 2.80, 95% CI = 2.37, 3.31), while children with ≥ 4 h/day screen time had reduced odds (OR = 0.39, 95% CI = 0.29, 0.52) of bedtime regularity (Table 4).

### Bedtime regularity in typically developing (TD) children

In the CRF model, factors most important to bedtime regularity in TD children included adequate sleep duration (VIMP = 5.75), family meals (3.68), difficulty making/keeping friends (VIMP = 3.23), age (3.16), and depression (2.99) (Fig. 1, Supplemental Table 4).

Logistic regression showed children with adequate sleep had 2.40 times higher odds of bedtime regularity (95% CI = 2.30, 2.52). Daily family meals were associated with three-fold increased odds (OR = 3.02, 95% CI = 2.74, 3.34), while those with high difficulty making/keeping friends had 52% lower odds (OR = 0.48, 95% CI = 0.42, 0.54) of bedtime regularity (Table 4).

When comparing factors associated with bedtime regularity between the two groups, similar patterns emerged: both adequate sleep duration and daily family meals were significantly associated with higher odds of bedtime regularity in children with and without NDDs. The strength of these associations was comparable across groups—for adequate sleep, the odds ratios were 2.86 for children with NDDs and 2.40 for TD children; for family meals, 2.80 and 3.02, respectively. However, distinct barriers were also observed: high screen time was linked to lower odds of bedtime regularity in children with NDDs (OR = 0.39), whereas difficulty making or keeping friends was associated with lower odds only in TD children (OR = 0.48).

## Discussion

Findings from this investigation revealed that a greater proportion of children with NDDs had parent reports of inadequate sleep duration (34.0% vs. 30.9%) and irregular bedtimes (18.8% vs. 13.7%) compared to TD children. For both groups, having more family meals together and achieving healthy sleep in the other domain (duration or bedtime regularity) were significantly associated with better sleep outcomes. Additionally, current depressive symptoms were linked to lower odds of adequate sleep in TD children and reduced bedtime regularity in both groups. These findings identify potential intervention targets to improve sleep in children aged 9–17 years, with or without NDDs.

Across the multivariable models of parent-reported sleep duration and bedtime regularity, the "other" sleep health variable emerged as the most strongly associated independent variable with each outcome in the current samples. Buysee’s framework of sleep health conceptualizes sleep duration and regularity as distinct, but related constructs for adults [29], while Meltzer and colleagues posit that regularity of sleep (that encompasses bedtime regularity) is inherent to the broader category of sleep-related behaviors that promote or inhibit sleep health in pediatric populations [30]. From a clinical and public health perspective, these findings support the common practice of first-line sleep health interventions for both TD children and those with NDDs. Indeed, sleep hygiene and cognitive behavioral therapy for insomnia approaches provide psychoeducation about child sleep needs, and behavioral modification to promote consistent and, as needed, earlier bedtimes, to allow for sufficient sleep duration [31, 32].

Consistent with Meltzer and colleagues framework for pediatric sleep health that emphasizes the importance of sleep related behaviors [30], data from this investigation showed that family meals together on more days of the week increased the odds of parent-report adequate and regular sleep in both groups. These results align with an earlier daily-diary study conducted in a nationally representative sample of 2454 TD children that showed each additional hour spent eating family meals during weekdays was associated with a 0.37 hours increase in total sleep time for younger children and a 0.57 hours increase in total sleep time for older children [33]. Noteworthy about the current study is that it extends this evidence to children with NDDs.

Family environment factors such as family function, connectedness and routines may help explain the strong link between regular family meals and achieving parent-reported adequate and bedtime regularity in children with and without NDDs. Previous studies have found that more regular family meals are associated with higher family functioning [34], stronger family bonds and connectedness [35], and enhanced feelings of inclusion among children with intellectual disabilities [36]. A large body of literature has reliably shown that in families with high function, characterized by positive and stable parental behaviors and feelings of connectedness between children and parents, children tend to demonstrate fewer sleep disturbances and better sleep health [37]. Relatedly, regular family meals support a robust, predictable rhythmicity in the family home that is a cornerstone of resilience, stability, self-regulation and positive outcomes in children [38-41]. Homes with frequent family meals may be more likely to have other robust rhythms such as structured bedtime routines, which are strongly linked to healthier sleep behaviors in children and adolescents [22, 42-44]. Behavioral and communication problems that serve as a defining characteristic for many children with NDDs [45] can make it particularly challenging for parents of children with NDDs to establish family routines (e.g., bedtime routines, regular meal times). Data from the current study underscore the need for pediatric sleep health interventions to address family routines, which may be enhanced by better family functioning. In the context of families with children who have NDDs, these approaches may need to offer more intensive support and consideration of behavioral and communication challenges [46].

Depression was found to be associated with a reduced likelihood of parent reported adequate sleep and bedtime regularity in typically developing children, and bedtime regularity in those with NDDs. These data align with evidence showing a bi-directional association between depressive symptoms and sleep disturbances in children and adolescents [47]. For example, poor and disturbed sleep health in childhood has been shown to independently predict a greater likelihood of increased depressive symptoms and depression in adolescence [48, 49]. Conversely, depression is predictive of subsequent disturbed sleep [50]. This result that depression was associated with bedtime regularity has implications for chronotherapy. Regular sleep is a function of a robust circadian rhythm that regulates patterns of alertness and sleepiness in each 24-hour cycle. Individuals with an evening chronotype or greater eveningness have a propensity for completing the bolus of activity later in the day as well as having later bed- and wake-times. While a shift toward greater “eveningness” is developmentally appropriate in adolescents [51], there is evidence that depression during childhood predicts a greater preference for eveningness post-puberty [52]. Moreover, greater eveningness preference longitudinally predicts increases in depression symptoms [52]. As a manifestation of circadian disruption and abnormal circadian rhythms, delayed and irregular sleep is especially common in children with NDDs including autism and Down’s syndrome as compared to TD children [53, 54]. Mechanisms to explain the higher prevalence of circadian dysregulation in children with NDDs include altered melatonin-cortisol rhythms, genetic polymorphisms affecting clock genes, and differences in light sensitivity that impact circadian entrainment [55, 56]. Given the critical role of circadian stability in mood regulation, these findings reinforce the need to target sleep regularity and circadian alignment in pediatric sleep health interventions, particularly for children with depression and NDDs.

Findings from this study have clinical and public health implications for pediatric sleep health interventions targeting both TD children and those with NDDs. Given the higher prevalence of inadequate sleep duration and irregular bedtimes in children with NDDs, targeted interventions addressing behavioral and environmental factors, such as family routines and sleep hygiene, are essential [30]. The strong association between sleep duration and regularity underscores the need for comprehensive strategies prioritizing both aspects of sleep health [29]. Additionally, the link between family meal frequency and improved sleep in both cohorts highlights the role of structured routines in shaping sleep behaviors [57]. While both NDD and TD children showed similar patterns with family meals and sleep variables emerging as top predictors, some important differences suggest the need for tailored approaches. Food sufficiency appeared more prominently in NDD models, indicating that socioeconomic interventions addressing food security may be particularly important for this population, likely reflecting the additional economic burden faced by families requiring specialized care and services. Conversely, neighborhood social support was more influential for TD children, suggesting that community-level interventions may be less critical than family-level factors for children with developmental challenges. Incorporating family-based routine interventions may be beneficial for all children’s sleep health, regardless of NDD status [58]. The relationship between depression and poor sleep suggests integrating mental health support into sleep interventions [59]. Given circadian dysregulation’s role in sleep disturbances, particularly in children with NDDs, interventions emphasizing chronotherapy and melatonin regulation may be crucial [60]. These findings suggest that while universal family-centered approaches around meal routines may benefit all children, targeted interventions addressing specific socioeconomic needs may be necessary for optimal outcomes in NDD populations. Results from the current study converge with Meltzer and colleagues’ framework [30] and support multifaceted, family-centered, and targeted interventions that address sleep health.

Strengths of this study include its large, nationally representative sample, the inclusion of both TD and NDD children, and rigorous analytic methods. However, several limitations should be noted. First, the NSCH relies on parent reports, which may be subject to recall bias. For example, parent reports of sleep may not be as reliable as device estimates. Device estimates provide more precise information about sleep patterns, which may be particularly important in NDD populations who often experience complex sleep disturbances. Second, findings from this U.S.-based study may not generalize to children in other countries where cultural sleep practices, family structures, socioeconomic conditions, and healthcare systems differ substantially. Family meal practices, neighborhood characteristics, and healthcare access patterns vary significantly across cultures and economic contexts. Third, several important factors that could influence sleep outcomes were not captured in the NSCH dataset, including sensory processing differences (particularly relevant for NDD populations), specific medication effects, comorbid medical conditions, sleep environment factors (noise, light exposure, bedroom sharing), and cultural or religious practices around sleep and family routines. Fourth, NDDs were grouped together, limiting the ability to examine differences by specific diagnosis. Fifth, the cross-sectional design with data collected across different time periods (2016–2021) prevents conclusions about causality and does not account for the possibility for different patterns of sleep health and associated factors across the study periods. Last, the exclusion of participants with missing data (*n* = 37,201) may introduce selection bias.

In conclusion, this study shows differences in sleep health between TD children and those with NDDs, suggesting the need for targeted interventions. The strong association between sleep duration and bedtime regularity suggests that comprehensive strategies addressing both factors are essential. Findings also underscore the importance of family routines, particularly regular family meals, and potential social connectedness among family members in promoting healthy sleep behaviors. Moreover, the link between depression and poor sleep highlights the need to integrate mental health support into sleep interventions. Directions for future research in this area include the conduct of prospective, longitudinal studies, using culturally and regionally diverse samples to ascertain the temporal associations of key determinants of device-estimated sleep behaviors. Studies that explore tailored approaches that incorporate chronotherapy and family-centered interventions to improve sleep health in children, particularly those with NDDs, are also warranted.

## Supplementary Material

Supplemental Tables

**Supplementary Information** The online version contains supplementary material available at https://doi.org/10.1007/s12519-025-00964-w.

## Figures and Tables

**Table 1:** Participant characteristics and bivariate associations with sleep outcomes in children with a neurodevelopmental disorder (*N* = 16,161)

Variables	Overall (*n*, %)	Adequate sleep duration			Bedtime regularity		
	
		No (*n* = 5493; 34.0%)	Yes (*n* = 10,668; 66.0%)	Test statistics	No (*n* = 3032; 18.8%)	Yes (*n* = 13,129; 80.2%)	Test statistics
**Dependent variables**							
Adequate sleep duration (*n*, %)				NA			718.06 (d = 0.54)
No	5493 (34.0)	5493 (100.0)	NA		1661 (54.8)	3832 (29.2)	
Yes	10,668 (66.0)	0 (0.0)	10,668 (100.0)		1371 (45.2)	9297 (70.8)	
Bedtime regularity (*n*, %)				718.06^†^ (d = 0.43)			NA
No	3032 (18.8)	1661 (30.2)	1371 (12.9)		3032 (100.0)	0 (0.0)	
Yes	13,129 (81.2)	3832 (69.8)	9297 (87.1)		0 (0.0)	13,129 (100.0)	
**Independent variables**							
**Individual level**							
Age (y; M, SD)	13.71 (2.28)	13.55 (2.44)	13.78 (2.20)	1.05^†^ (d = 0.10)	14.39 (2.19)	13.55 (2.28)	0.85^†^ (d = 0.38)
Sex (*n*, %)				0.58 (d = 0.01)			10.91^†^ (d = 0.07)
Male	10,525 (65.1)	3555 (64.7)	6970 (65.3)		1896 (62.5)	8629 (65.7)	
Female	5636 (10.5)	1938 (35.3)	3698 (34.7)		1136 (37.5)	4500 (34.3)	
Race/Ethnicity (*n*, %)				99.00^†^ (d = 0.16)			114.35^†^ (d = 0.20)
Hispanic	1693 (10.5)	619 (11.3)	1074 (10.1)		381 (12.6)	1312 (10.0)	
White, non-Hispanic	11,970 (74.1)	3884 (70.7)	8086 (75.8)		2056 (67.8)	9914 (75.5)	
Black, non-Hispanic	954 (5.9)	456 (8.3)	498 (4.7)		285 (9.4)	669 (5.1)	
Other/multiracial, non-Hispanic	1544(9.6)	534 (9.7)	1010 (9.5)		310 (10.2)	1234 (9.4)	
Family income [federal poverty level (FPL); *n*, %]				145.11^†^ (d = 0.20)			181.73^†^ (d = 0.26)
0–99	1989 (12.3)	858 (15.6)	1131 (10.6)		566 (18.7)	1423 (10.8)	
100–199	2701 (16.7)	1021 (18.6)	1680 (15.7)		570 (18.8)	2131 (16.2)	
200–399	4846 (30.0)	1644 (29.9)	3202 (30.0)		872 (28.8)	3974 (30.3)	
≥ 400	6625 (41.0)	1970 (35.9)	4655 (43.6)		1024 (33.8)	5601 (42.7)	
Food insufficiency (*n*, %)				210.87^†^ (d = 0.24)			312.98^†^ (d = 0.34)
We could always afford to eat (“good nutritious meals” ^‡^)	10,785 (66.7)	3264 (59.4)	7521 (70.5)		1641 (54.1)	9144 (69.6)	
We could always afford enough to eat, but not always the kinds of foods we should eat	4354 (26.9)	1762 (32.1)	2592 (24.3)		1051 (34.7)	3303 (25.2)	
We sometimes could not afford to eat	849 (5.3)	391 (7.1)	458 (4.3)		285 (9.4)	564 (4.3)	
Often, we could not afford to eat	173 (1.1)	76 (1.4)	97 (0.9)		55 (1.8)	118 (0.9)	
Physical activity levels, ages 6–17 y (days per week; *n*, %)				55.83^†^ (d = 0.13)			362.28^†^ (d = 0.37)
0	2522 (15.6)	965 (17.6)	1557 (14.6)		785 (25.9)	1737 (13.2)	
1–3	7071 (43.8)	2490 (45.3)	4581 (42.9)		1345 (44.4)	5726 (43.6)	
4–6	4049 (25.1)	1215 (22.1)	2834 (26.6)		553 (18.2)	3496 (26.6)	
Every day	2519 (15.6)	823 (15.0)	1696 (15.9)		349 (11.5)	2170 (16.5)	
Screen time (h per day; *n*, %)				28.27^†^ (d = 0.09)			289.00^†^ (d = 0.36)
≤ 1	679 (4.2)	196 (3.6)	483 (4.5)		52 (1.7)	627 (4.8)	
1–3	6515 (40.3)	2099 (38.2)	4416 (41.4)		889 (29.3)	5626 (42.9)	
≥ 4	8967 (55.5)	3198 (58.2)	5769 (54.1)		2091 (69.0)	6876 (52.4)	
Ever told child has depression (*n*, %)	12,578 (77.8)			37.10^†^ (d = 0.10)			442.86^†^ (d = 0.40)
Do not have condition	573 (3.5)	4132 (75.2)	8446 (79.2)		1934 (63.8)	10,644 (81.1)	
Ever told, but do not currently have	3010 (18.6)	196 (3.6)	377 (3.5)		141 (4.7)	432 (3.3)	
Currently have	12,578 (77.8)	1165 (21.2)	1845 (17.3)		957 (31.6)	2053 (15.6)	
**Social level**							
Maternal mental health (*n*, %)				148.92^†^ (d = 0.20)			405.19^†^ (d = 0.39)
Excellent or very good	10,221 (63.2)	3136 (57.1)	7085 (66.4)		1493 (49.2)	8728 (66.5)	
Good	4345 (26.9)	1665 (30.3)	2680 (25.1)		999 (32.9)	3346 (25.5)	
Fair or poor	1595 (9.9)	692 (12.6)	903 (8.5)		540 (17.8)	1055 (8.0)	
Family eats meals together (days per week; *n*, %)				212.28^†^ (d = 0.24)			578.89^†^ (d = 0.46)
No	903 (5.6)	418 (7.6)	485 (4.5)		363 (12.0)	540 (4.1)	
1–3	5006 (31.0)	1999 (36.4)	3007 (28.2)		1261 (41.6)	3745 (28.5)	
4–6	5342 (33.1)	1605 (29.2)	3737 (35.0)		752 (24.8)	4590 (35.0)	
Every day	4910 (30.4)	1471 (26.8)	3439 (32.2)		656 (21.6)	4254 (32.4)	
Difficulty making/keeping friends (*n*, %)				7.81* (d = 0.05)			60.93^†^ (d = 0.16)
No difficulty	7075 (43.8)	2346 (42.7)	4729 (44.3)		1140 (37.6)	5935 (45.2)	
A little difficulty	5678 (35.1)	2010 (36.6)	3668 (34.4)		1151 (38.0)	4527 (34.5)	
A lot of difficulty	3408 (21.1)	1137 (20.7)	2271 (21.3)		741 (24.4)	2667 (20.3)	
Someone smokes inside the home (*n*, %)				88.98* (d = 0.15)			148.38^†^ (d = 0.23)
No one smokes in the household	13,204 (81.7)	4280 (77.9)	8924 (83.7)		2266 (74.7)	10,938 (83.3)	
Someone smokes, not inside the house	2481 (15.4)	989 (18.0)	1492 (14.0)		602 (19.9)	1879 (14.3)	
Someone smokes inside the house	476 (2.9)	224 (4.1)	252 (2.4)		164 (5.4)	312 (2.4)	
Parental aggravation (w/parenting), in the last month (*n*, %)				4.47* (d = 0.04)			111.38^†^ (d = 0.20)
Parent usually/always feels aggravation from parenting	2972 (18.4)	1060 (19.3)	1912 (17.9)		761 (25.1)	2211 (16.8)	
Parent seldom feels aggravation from parenting	13,189 (81.6)	4433 (80.7)	8756 (82.1)		2271 (74.9)	10,918 (83.2)	
**Environmental level**							
Neighborhood safety (*n*, %)				52.08^†^ (d = 0.12)			112.37^†^ (d = 0.21)
Definitely agree	11,003 (68.1)	3549 (64.6)	7454 (69.9)		1841 (60.7)	9162 (69.8)	
Somewhat agree	4453 (27.6)	1650 (30.0)	2803 (26.3)		986 (32.5)	3467 (26.4)	
Somewhat/Definitely disagree	705 (4.4)	294 (5.4)	411 (3.9)		205 (6.8)	500 (3.8)	
Detracting neighborhood elements (*n*, %)				35.90^†^ (d = 0.10)			65.93^†^ (d = 0.16)
0	12,586 (77.9)	4144 (75.4)	8442 (79.1)		2198 (72.5)	10,388 (79.1)	
1	2209 (13.7)	819 (14.9)	1390 (13.0)		507 (16.7)	1702 (13.0)	
2	787 (4.9)	284 (5.2)	503 (4.7)		177 (5.8)	610 (4.6)	
3	579 (3.6)	246 (4.5)	333 (3.1)		150 (4.9)	429 (3.3)	
Neighborhood social support (*n*, %)				75.21^†^ (d = 0.15)			199.28^†^ (d = 0.29)
Lives in a supportive neighborhood	9052 (56.0)	2817 (51.3)	6235 (58.4)		1350 (44.5)	7702 (58.7)	
Does not live in a supportive neighborhood	7109 (44.0)	2676 (48.7)	4433 (41.6)		1682 (55.5)	5427 (41.3)	
Neighborhood amenities (*n*, %)				7.076 (d = 0.04)			12.85* (d = 0.07)
Neighborhood does not contain any amenities	2068 (12.8)	734 (13.4)	1334 (12.5)		419 (13.8)	1649 (12.6)	
Neighborhood contains 1 amenity	1963 (12.1)	693 (12.6)	1270 (11.9)		381 (12.6)	1582 (12.0)	
Neighborhood contains 2 amenities	2903 (18.0)	998 (18.2)	1905 (17.9)		575 (19.0)	2328 (17.7)	
Neighborhood contains 3 amenities	3757 (23.2)	1272 (23.2)	2485 (23.3)		708 (23.4)	3049 (23.2)	
Neighborhood contains all 4 amenities listed	5470 (33.8)	1796 (32.7)	3674 (34.4)		949 (31.3)	4521 (34.4)	
Access to family centered care in the past 12 months (*n*, %)				49.69^†^ (d = 0.12)			186.51 (d = 0.26)
Did not have health care visit	1667 (10.3)	611 (11.1)	1056 (9.9)		374 (12.3)	1293 (9.8)	
Received family centered care	12,521 (77.5)	4088 (74.4)	8433 (79.0)		2086 (68.8)	10,435 (79.5)	
Did not receive family centered care	1973 (12.2)	794 (14.5)	1179 (11.1)		572 (18.9)	1401 (10.7)	

* *P* > 0.05

† *P* > 0.01

‡ Verbiage added in the 2020–2021 NSCH that was not included in prior year’s surveys *NA* not available

**Table 2:** Participant characteristics and bivariate associations with sleep outcomes in typically developing children (*N* = 69,853)

Variables	Overall (*n*, %)	Adequate sleep duration			Bedtime regularity		
	
		No (*n* = 21,598; 30.9%)	Yes (*n* = 48,255; 69.1%)	Test statistics	No (*n* = 9593; 13.7%)	Yes (*n* = 60,260; 86.3%)	Test statistics
**Dependent variables**							
Adequate sleep duration (*n*, %)				NA			1891.33^†^ (d = 0.47)
No	21,598 (30.9)	48,255 (100)	0 (0.0)		4975 (50.0)	16,803 (27.9)	
Yes	48,255 (69.1)	0 (0.0)	48,255 (100.0)		4798 (50.0)	43,457 (72.1)	
Bedtime regularity (*n*, %)				1891.33^†^ (d = 0.34)			NA
No	9593 (13.7)	4795 (22.2)	4798 (9.9)		60,260 (100.00)	0 (0.0)	
Yes	60,260 (86.3)	16,803 (77.8)	43,457 (90.1)		0 (0.0)	60,260 (100.0)	
**Independent variables-individual level**							
Age (y; M, SD)	13.83 (2.28)	13.80 (2.44)	13.84 (2.21)	1.006	14.53 (2.13)	13.71 (2.29)	0.85^†^ (d = 0.37)
Sex (*n*, %)				39.41^†^ (d = 0.05)			30.13^†^ (d = 0.06)
Male	33,583 (48.1)	10,000 (46.3)	23,583 (48.9)		4362 (45.5)	29,221 (48.5)	
Female	36,270 (51.9)	11,598 (53.7)	24,672 (51.1)		5231 (54.5)	31,039 (51.5)	
Race/Ethnicity (*n*, %)				566.46^†^ (d = 0.19)			634.06^†^ (d = 0.26)
Hispanic	8207 (11.7)	2860 (13.2)	5347 (11.1)		1423 (14.8)	6784 (11.3)	
White, non-Hispanic	48,843 (69.9)	14,038 (65.0)	34,805 (72.1)		5828 (60.8)	43,015 (71.4)	
Black, non-Hispanic	3882 (5.6)	1763 (8.2)	2119 (4.4)		954 (9.9)	2928 (4.9)	
Other/multiracial, non-Hispanic	8921 (12.8)	2937 (13.6)	5984 (12.4)		1388 (14.5)	7533 (12.5)	
Federal poverty level [(FPL); *n*, %)]				482.79^†^ (d = 0.18)			683.18^†^ (d = 0.28)
0–99	6192 (8.9)	2323 (10.8)	3869 (8.0)		1322 (13.8)	4870 (8.1)	
100–199	9866 (14.1)	3542 (16.4)	6324 (13.1)		1773 (18.5)	8093 (13.4)	
200–399	21,468 (30.7)	6966 (32.3)	14,502 (30.1)		2996 (31.2)	18,472 (30.7)	
≥ 400	32,327 (46.3)	8767 (40.6)	23,560 (48.8)		3502 (36.5)	28,825 (47.8)	
Family structure (*n*, %)				400.859^†^ (d = 0.16)			465.397^†^ (d = 0.23)
Two parents, currently married	54,954 (78.7)	15,992 (74.0)	38,962 (80.7)		6749 (70.4)	48,205 (80.0)	
Two parents, not currently married	3673 (5.3)	1399 (6.5)	2274 (4.7)		656 (6.8)	3017 (5.0)	
Single parent	11,146 (16.0)	4182 (19.4)	6964 (14.4)		2175 (22.7)	8971 (14.9)	
Other family structure	80 (0.1)	25 (0.1)	55 (0.1)		13 (0.1)	67 (0.1)	
Food sufficiency (*n*, %)				802.16^†^ (d = 0.23)			887.121^†^ (d = 0.31)
We could always afford to eat (“good nutritious meals”^†^)	53,950 (77.2)	15,253 (70.6)	38,697 (80.2)		6314 (65.8)	47,636 (79.1)	
We could always afford enough to eat, but not always the kinds of foods we should eat	13,905 (19.9)	5460 (25.3)	8445 (17.5)		2753 (28.7)	11,152 (18.5)	
We sometimes could not afford to eat	1722 (2.5)	748 (3.5)	974 (2.0)		456 (4.8)	1266 (2.1)	
Often, we could not afford to eat	276 (0.4)	137 (0.6)	139 (0.3)		70 (0.7)	206 (0.3)	
BMI (*n*, %)			387.12^†^ (d = 0.16)			188.09^†^ (d = 0.14)
Underweight	4160 (6.0)	1196 (5.5)	2964 (6.1)		509 (5.3)	3651 (6.1)	
Healthy weight	47,209 (67.6)	13,700 (63.4)	33,509 (69.4)		6097 (63.6)	41,112 (68.2)	
Overweight	10,019 (14.3)	3408 (15.8)	6611 (13.7)		1436 (15.0)	8583 (14.2)	
Obese	8465 (12.1)	3294 (15.3)	5171 (10.7)		1551 (16.2)	6914 (11.5)	
Physical activity, (days per week; *n*, %)				159.20^†^ (d = 0.10)			1243.33^†^ (d = 0.36)
0	6732 (9.6)	2412 (11.2)	4320 (9.0)		1756 (18.3)	4976 (8.3)	
1–3	28,121 (40.3)	9040 (41.9)	19,081 (39.5)		4213 (43.9)	23,908 (39.7)	
4–6	22,828 (32.7)	6680 (30.9)	16,148 (33.5)		2409 (25.1)	20,419 (33.9)	
Every day	12,172 (17.4)	3466 (16.0)	8706 (18.0)		1215 (12.7)	10,957 (18.2)	
Screen time (hours per day; *n*, %)				207.85^†^ (d = 0.12)			925.65^†^ (d = 0.35)
≤ 1	3221 (4.6)	758 (3.5)	2463 (5.1)		228 (2.4)	2993 (5.0)	
1–3	31,326 (44.8)	9141 (42.3)	22,185 (46.0)		3149 (32.8)	28,177 (46.8)	
≥ 4	35,306 (50.5)	11,699 (54.2)	23,607 (48.9)		6216 (64.8)	29,090 (48.3)	
Ever told child has depression (*n*, %)				187.21^†^ (d = 0.11)			975.20^†^ (d = 0.29)
Do not have condition	65,764 (94.1)	19,951 (92.4)	45,813 (94.9)		8380 (87.4)	57,384 (95.2)	
Ever told, but do not currently have	973 (1.4)	354 (1.6)	619 (1.3)		226 (2.4)	747 (1.2)	
Currently have	3116 (4.5)	1293 (6.0)	1823 (3.8)		987 (10.3)	2129 (3.5)	
**Social level**							
Maternal mental health (*n*, %)				512.59^†^ (d = 0.18)			1250.42^†^ (d = 0.36)
Excellent or very good	53,782 (77.0)	15,491 (71.7)	38,291 (79.4)		6125 (63.8)	47,657 (79.1)	
Good	12,953 (18.5)	4812 (22.3)	8141 (16.9)		2573 (26.8)	10,380 (17.2)	
Fair or poor	3118 (4.5)	1295 (6.0)	1823 (3.8)		895 (9.3)	2223 (3.7)	
Family eats meals together (days per week; n, %)				951.16^†^ (d = 0.25)			1666.23^†^ (d = 0.42)
0	2520 (3.6)	1136 (5.3)	1384 (2.9)		823 (8.6)	1697 (2.8)	
1–3	20,631 (29.5)	7716 (35.7)	129,15 (26.8)		3891 (40.6)	16,740 (27.8)	
4–6	25,225 (36.1)	7114 (32.9)	18,111 (37.5)		2805 (29.2)	22,420 (37.2)	
Every day	21,477 (30.7)	5632 (26.1)	15,845 (32.8)		2074 (21.6)	19,403 (32.2)	
Difficulty making/keeping friends (*n*, %)				59.80^†^ (d = 0.06)			681.47^†^ (d = 0.26)
No difficulty	56,396 (80.7)	17,083 (79.1)	39,313 (81.5)		6910 (72.0)	49,486 (82.1)	
A little difficulty	12,005 (17.2)	3988 (18.5)	8017 (16.6)		2247 (23.4)	9758 (16.2)	
A lot of difficulty	1,452 (2.1)	527 (2.4)	925 (1.9)		436 (4.5)	1016 (1.7)	
Someone smokes Inside the home (*n*, %)				219.75^†^ (d = 0.12)			242.45^†^ (d = 0.16)
No one smokes in the household	60,927 (87.2)	18,263 (84.6)	42,664 (88.4)		7939 (82.8)	52,988 (87.9)	
Someone smokes, not inside the house	7,767 (11.1)	2835 (13.1)	4932 (10.2)		1367 (14.2)	6400 (10.6)	
Someone smokes inside the house	1,159 (1.7)	500 (2.3)	659 (1.4)		287 (3.0)	872 (1.4)	
Parental aggravation (w/parenting), in the last month (*n*, %)				96.10^†^ (d = 0.08)			737.92^†^ (d = 0.23)
Parent usually/always feels aggravation from parenting	1701 (2.4)	711 (2.3)	990 (2.1)		615 (6.4)	1086 (1.8)	
Parent seldom feels aggravation from parenting	68,152 (97.6)	20,887 (96.7)	47,265 (97.9)		8978 (93.6)	59,174 (98.2)	
**Environmental level**							
Neighborhood safety (*n*, %)				410.99^†^ (d = 0.16)			556.92^†^ (d = 0.24)
Definitely agree	51,321 (73.5)	14,799 (68.5)	36,522 (75.7)		6203 (64.7)	45,118 (74.9)	
Somewhat agree	16,821 (24.1)	6094 (28.2)	10,727 (22.2)		2932 (30.6)	13,889 (23.0)	
Somewhat/Definitely disagree	1711 (2.4)	705 (3.3)	1006 (2.1)		458 (4.8)	1253 (2.1)	
Detracting neighborhood elements (*n*, %)				157.68^†^ (d = 0.10)			320.95* (d = 0.19)
0	56,224 (80.5)	16,802 (77.8)	39,422 (81.7)		7107 (74.1)	49,117 (81.5)	
1	9110 (13.0)	3136 (14.5)	5974 (12.4)		1568 (16.3)	7542 (12.5)	
2	2765 (4.0)	980 (4.5)	1785 (3.7)		534 (5.6)	2231 (3.7)	
3	1754 (2.5)	680 (3.1)	1074 (2.2)		384 (4.0)	1370 (2.3)	
Neighborhood social support (*n*, %)				469.24^†^ (d = 0.18)			841.21^†^ (d = 0.32)
Lives in a supportive neighborhood	44,493 (63.4)	12,484 (57.8)	32,009 (66.3)		4841 (50.5)	39,652 (65.8)	
Does not live in a supportive neighborhood	25,360 (36.3)	9114 (42.2)	16,246 (33.7)		4752 (49.5)	20,608 (34.2)	
Neighborhood amenities (*n*, %)				64.99^†^ (d = 0.07)			95.28^†^ (d = 0.11)
Neighborhood does not contain any amenities	8626 (12.3)	2799 (13.0)	5827 (12.1)		1235 (12.9)	7391 (12.3)	
Neighborhood contains 1 amenity	7638 (10.9)	2465 (11.4)	5173 (10.7)		1196 (12.5)	6442 (10.7)	
Neighborhood contains 2 amenities	11,967 (17.1)	3891 (18.0)	8076 (16.7)		1825 (19.0)	10,142 (16.8)	
Neighborhood contains 3 amenities	15,712 (22.5)	4870 (22.5)	10,842 (22.5)		2153 (22.4)	13,559 (22.5)	
Neighborhood contains all 4 amenities listed	25,910 (37.1)	7573 (35.1)	18,337 (38.0)		3184 (33.2)	22,726 (37.7)	
Access to family centered care in the past 12 months (*n*, %)				110.38^†^ (d = 0.09)			597.34^†^ (d = 0.25)
Did not have health care visit	12,191 (17.5)	3948 (18.3)	8243 (17.1)		1947 (20.3)	10,244 (17.0)	
Received family centered care	52,340 (74.9)	15,707 (72.7)	36,633 (75.9)		6387 (66.6)	45,953 (76.3)	
Did not receive family centered care	5322 (7.6)	1943 (9.0)	3379 (7.0)		1259 (13.1)	4063 (6.7)	

* *P* > 0.05

† *P* > 0.01. Verbiage added in the 2020–2021 NSCH that was not included in prior year’s surveys *NA* not available

**Table 3:** Multivariable logistic regression of adequate sleep duration in typically developing (TD) children with neurodevelopmental disorders (NDD)

Variables	NDD (*n* = 16,161) OR (95% CI)	TD (*n* = 69,853) OR (95% CI)
Age	1.09 (1.08, 1.11)	1.05 (1.04, 1.06)
Bedtime regularity	2.78 (2.55, 3.02)	2.40 (2.30, 2.52)
Food sufficiency		NA
We could always afford to eat good nutritious meals	REF	–
We could always afford enough to eat, but not always the kinds of foods we should eat	0.74 (0.685, 0.798)	–
We sometimes could not afford to eat	0.65 (0.56, 0.75)	–
Often, we could not afford to eat	0.69 (0.50, 0.94)	–
Smoke inside the home		N/A
No one smokes in the household	REF	–
Someone smokes, not inside the house	0.85 (0.78, 0.94)	–
Someone smokes inside the house	0.74 (0.61, 0.90)	–
Frequency of family meals together		
No meals	REF	REF
1–3 d/wk	1.10 (0.943, 1.271)	1.22 (1.12, 1.33)
4–6 d/wk	1.54 (1.33, 1.80)	1.76 (1.62, 1.92)
Every day of week	1.63 (1.40, 1.90)	1.97 (1.80, 2.15)
Depression	NA	
Do not have condition	–	REF
Ever told, but do not currently have	–	0.85 (0.74, 0.97)
Currently have	–	0.76 (0.70, 0.82)
Parental aggravation (w/parenting) in the last month (2 = High vs. 1 = Low)	–	1.18 (1.06, 1.31)

*REF* reference category, *OR* odds ratio, *CI* confidence interval, *NA* non-applicable

**Table 4:** Multivariable logistic regression of bedtime regularity in children with NDD and TD

Variables	NDD (*n* = 16,161) OR (95% CI)	TD (*n* = 69,853) OR (95% CI)
Adequate sleep	2.86 (2.63, 3.12)	2.40 (2.30, 2.52)
Age	0.88 (0.86, 0.90)	0.88 (0.87, 0.89)
Depression		
Do not have condition	REF	REF
Ever told, but do not currently have	0.69 (0.57, 0.86)	0.72 (0.62, 0.85)
Currently have	0.50 (0.45, 0.55)	0.53 (0.49, 0.58)
Screen time		NA
≤ 1 h/d	REF	–
1–3 h/d	0.61 (0.45, 0.82)	–
≥ 4 h/d	0.39 (0.29, 0.52)	–
Frequency of family meals together		
No meals	REF	REF
1–3 d/wk	1.73 (1.47, 2.02)	1.81 (1.65, 1.99)
4–6 d/wk	2.89 (2.46, 3.41)	2.85 (2.58, 3.13)
Every day of week	2.80 (2.37, 3.31)	3.02 (2.74, 3.34)
Difficulty making/Keeping friends	NA	
No difficulty	–	REF
A little difficulty	–	0.69 (0.65, 0.73)
A lot of difficulty	–	0.48 (0.42, 0.54)

*NDD* neurodevelopmental disorders, *TD* typically developing, *REF* reference category, *OR* odds ratio, *CI* confidence interval, *NA* non-applicable

## Data Availability

The National Survey of Children's Health (NSCH) data are publicly available.
