# Supplementary material for: Multilevel factors associated with sleep duration and bedtime regularity in U.S. children with and without neurodevelopmental disorders: a nationally representative study
Source: World J Pediatr. 2025 Oct 3;21(11):1120–39. doi: 10.1007/s12519-025-00964-w (PMC12627111; doi:10.1007/s12519-025-00964-w)
Supplement: Supplementary file 1 [file 12519_2025_964_MOESM1_ESM.docx]

**Supplemental Table 1:** Variables of most importance associated with adequate sleep duration in children with neurodevelopmental disorders 

| **Variable** | **VIMP** **(95% Confidence Interval Lower, Upper)** |
| --- | --- |
| Age | 7.79 (3.64, 5.93) |
| Bedtime regularity | 7.17 (5.89, 8.45) |
| Food Sufficiency | 2.10 (0.58, 3.63) |
| Smoke Inside the Home | 1.72 (0.45, 2.99) |
| Family Eats Meals Together | 1.71 (0.47, 2.94) |
| Race | 1.59 (0.22, 2.95) |
| Detracting Neighborhood Elements | 1.52 (0.69, 2.36) |
| Screen Time | 1.20 (0.00, 2.4) |
| Physical Activity | 1.04 (0.10, 1.87) |
| Neighborhood Amenities | 0.93 (0.23, 1.64) |
| Depression | 0.90 (-0.15, 1.95) |
| Neighborhood Safety | 0.62 (-0.24, 1.48) |
| Family Income | 0.61 (-0.42, 1.63) |
| Mother Maternal Health | 0.60 (-0.7, 1.89) |
| Family Centered Care in the past 12-months | 0.54 (-0.37, 1.46) |
| Difficulty Making/Keeping Friends | 0.31 (-0.40, 1.03) |
| Sex | 0.26 (-0.44, 0.97) |
| Neighborhood Social Support | 0.15 (-1.33, 1.63) |
| Child is Bullied by Others (in the past 12 months***; children aged 6-17) | -0.05 (-0.62, 0.52) |
| Parental Aggravation (w/ parenting) in the last month | 0.03 (-0.55, 0.61) |

**Supplemental Table 2:** Variables of most importance associated with bedtime regularity in children with neurodevelopmental disorders

| **Variable** | **VIMP (95% Confidence Interval Lower, Upper)** |
| --- | --- |
| Adequate Sleep | 6.52 (4.42, 8.61) |
| Age | 3.59 (1.79, 5.38) |
| Depression | 2.85 (0.87, 4.82) |
| Screen Time | 2.72 (0.65, 4.80) |
| Family Eats Meals Together | 2.37 (1.58, 3.17) |
| Food Sufficiency | 1.81 (0.14, 3.48) |
| Smoke Inside the Home | 1.79 (0.79, 2.80) |
| Mother Maternal Health | 1.76 (0.22, 3.29) |
| Family Income | 1.61 (0.57, 2.66) |
| Physical Activity | 1.56 (0.28, 2.84) |
| Race | 1.34 (0.03, 2.45) |
| Detracting Neighborhood Elements | 1.26 (0.22, 2.31) |
| Family Centered Care in the past 12-months | 1.03 (-0.23, 2.29) |
| Neighborhood Amenities | 0.88 (0.00, 1.76) |
| Difficulty Making/Keeping Friends | 0.41 (-0.34, 1.17) |
| Neighborhood Safety | 0.41 (-0.51, 1.33) |
| Neighborhood Social Support | 0.36 (-0.88, 1.60) |
| Child is Bullied by Others (in the past 12 months***; children aged 6-17) | 0.21 (-0.43, 0.84) |
| Parental Aggravation (w/ parenting) in the last month | 0.21 (-0.44, 0.86) |
| Sex | -0.01 (-0.65, 0.58) |

**Supplemental Table 3:** Variables of most importance associated with adequate sleep duration in typically developing children 

| **Variable** | **VIMP (95% Confidence Interval Lower, Upper)** |
| --- | --- |
| Bedtime regularity | 5.16 (4.36, 5.96) |
| Age | 4.19 (3.48, 4.89) |
| Depression | 3.12 (1.99, 4.24) |
| Family Eats Meals Together | 2.37 (1.58, 3.17) |
| Parental Aggravation (w/ parenting) in the last month | 1.79 (0.94, 2.64) |
| Food Sufficiency | 1.59 (0.58, 2.60) |
| Smoke Inside the Home | 1.51 (0.51, 2.51) |
| Race | 1.39 (0.71, 1.97) |
| Detracting Neighborhood Elements | 1.31 (0.65, 1.31) |
| Difficulty Making/Keeping Friends | 1.18 (0.55, 1.82) |
| Family Income | 0.92 (0.26, 1.59) |
| Screen Time | 0.91 (0.27, 1.55) |
| Mother Maternal Health | 0.83 (0.05, 1.62) |
| Physical Activity | 0.58 (-0.03, 1.20) |
| Neighborhood Safety | 0.48 (-0.36, 1.33) |
| Sex | -0.41 (0.15, 0.70) |
| Child is Bullied by Others (in the past 12 months***; children aged 6-17) | -0.33 (0.17, 0.67) |
| Family Centered Care in the past 12-months | 0.31 (-0.20, 0.82) |
| Neighborhood Amenities | 0.25 (-0.27, 0.76) |
| Neighborhood Social Support | 0.22 (-0.74, 1.18) |

**Supplemental Table 4:** Variables of most Importance associated with bedtime regularity in typically developing children

| **Variable** | **VIMP (95% Confidence Interval Lower, Upper)** |
| --- | --- |
| Adequate Sleep | 5.75 (4.84, 6.66) |
| Family Eats Meals Together | 3.68 (2.50, 4.87) |
| Difficulty Making/Keeping Friends | 3.23 (1.93, 4.53) |
| Age | 3.16 (1.96, 4.36) |
| Depression | 2.99 (1.26, 4.72) |
| Neighborhood Detracting Elements | 2.46 (1.64, 3.27) |
| Mother Maternal Health | 2.31 (1.11, 3.5) |
| Screen Time | 2.10 (0.96, 3.24) |
| Smoke Inside the Home | 2.10 (1.32, 2.87) |
| Food Sufficiency | 1.96 (0.92, 3.01) |
| Race | 1.82 (0.91, 2.72) |
| Parental Aggravation (w/ parenting) in the last month | 1.80 (0.80, 2.80) |
| Family Income | 1.79 (0.87, 2.72) |
| Physical Activity | 1.69 (0.73, 2.65) |
| Family Centered Care in the past 12-months | 1.51 (0.73, 2.29) |
| Neighborhood Safety | 1.45 (0.71, 2.18) |
| Neighborhood Amenities | 0.87 (0.27, 1.47) |
| Neighborhood Social Support | 0.49 (-0.82, 1.81) |
| Child is Bullied by Others (in the past 12 months***; children aged 6-17) | -0.37 (-0.26, 1.00) |
| Sex | 0.32 (-0.16, 0.80) |
